# Sublingual Misoprostol versus Intramuscular Oxytocin for Prevention of Postpartum Hemorrhage in Uganda: A Double-Blind Randomized Non-Inferiority Trial

**DOI:** 10.1371/journal.pmed.1001752

**Published:** 2014-11-04

**Authors:** Esther C. Atukunda, Mark J. Siedner, Celestino Obua, Godfrey R. Mugyenyi, Marc Twagirumukiza, Amon G. Agaba

**Affiliations:** 1 Mbarara University of Science and Technology, Mbarara, Uganda; 2 Department of Medicine, Massachusetts General Hospital, Boston, Massachusetts, United States of America; 3 Department of Pharmacology and Therapeutics, School of Biomedical Sciences, College of Health Sciences, Makerere University, Kampala, Uganda; 4 Department of Pharmacology, Faculty of Medicine and Health Sciences, Ghent University, Ghent, Belgium; The University of Adelaide, Australia

## Abstract

**Background:**

Postpartum hemorrhage (PPH) is a leading cause of maternal death in sub-Saharan Africa. Although the World Health Organization recommends use of oxytocin for prevention of PPH, misoprostol use is increasingly common owing to advantages in shelf life and potential for sublingual administration. There is a lack of data about the comparative efficacy of oxytocin and sublingual misoprostol, particularly at the recommended dose of 600 µg, for prevention of PPH during active management of labor.

**Methods and Findings:**

We performed a double-blind, double-dummy randomized controlled non-inferiority trial between 23 September 2012 and 9 September 2013 at Mbarara Regional Referral Hospital in Uganda. We randomized 1,140 women to receive 600 µg of misoprostol sublingually or 10 IU of oxytocin intramuscularly, along with matching placebos for the treatment they did not receive. Our primary outcome of interest was PPH, defined as measured blood loss ≥500 ml within 24 h of delivery. Secondary outcomes included measured blood loss ≥1,000 ml; mean measured blood loss at 1, 2, and 24 h after delivery; death; requirement for blood transfusion; hemoglobin changes; and use of additional uterotonics.

At 24 h postpartum, primary PPH occurred in 163 (28.6%) participants in the misoprostol group and 99 (17.4%) participants in the oxytocin group (relative risk [RR] 1.64, 95% CI 1.32 to 2.05, *p*<0.001; absolute risk difference 11.2%, 95% CI 6.44 to 16.1). Severe PPH occurred in 20 (3.6%) and 15 (2.7%) participants in the misoprostol and oxytocin groups, respectively (RR 1.33, 95% CI 0.69 to 2.58, *p* = 0.391; absolute risk difference 0.9%, 95% CI −1.12 to 2.88). Mean measured blood loss was 341.5 ml (standard deviation [SD] 206.2) and 304.2 ml (SD 190.8, *p* = 0.002) at 2 h and 484.7 ml (SD 213.3) and 432.8 ml (SD 203.5, *p*<0.001) at 24 h in the misoprostol and oxytocin groups, respectively. There were no significant differences between the two groups in any other secondary outcomes. Women in the misoprostol group more commonly experienced shivering (RR 1.91, 95% CI 1.65 to 2.21, *p*<0.001) and fevers (RR 5.20, 95% CI 3.15 to 7.21, *p* = 0.005).

This study was conducted at a regional referral hospital with capacity for emergency surgery and blood transfusion. High-risk women were excluded from participation.

**Conclusions:**

Misoprostol 600 µg is inferior to oxytocin 10 IU for prevention of primary PPH in active management of labor. These data support use of oxytocin in settings where it is available. While not powered to do so, the study found no significant differences in rate of severe PPH, need for blood transfusion, postpartum hemoglobin, change in hemoglobin, or use of additional uterotonics between study groups. Further research should focus on clarifying whether and in which sub-populations use of oxytocin would be preferred over sublingual misoprostol.

**Trial registration:**

ClinicalTrials.gov NCT01866241

*Please see later in the article for the Editors' Summary*

## Abstract 2

In a double-blind randomized controlled trial, Esther Atukunda and colleagues evaluated whether sublingual misoprostol administered to women in labor was non-inferior to intramuscular oxytocin in preventing postpartum hemorrhage and reducing blood loss.

*Please see later in the article for the Editors' Summary*

## Editors' Summary

**Background:**

Every year, worldwide nearly 290,000 women die during pregnancy or labor or during the first six weeks after giving birth (the postpartum period). Almost all of these “maternal” deaths occur in low- or middle-income countries, and most are caused by a handful of preventable or treatable conditions—postpartum hemorrhage (severe bleeding from the uterus [womb] within 24 hours of childbirth), post-delivery infections, unsafe abortion, obstructive (difficult) labor, and blood pressure disorders during pregnancy. The leading cause of maternal deaths worldwide is postpartum hemorrhage, which is responsible for 25%–30% of all maternal deaths. Postpartum hemorrhage can be prevented by giving the mother an intramuscular injection of oxytocin, a hormone that stimulates uterine contractions and limits uterine bleeding, immediately after her child is born.

**Why Was This Study Done?:**

Unfortunately, oxytocin needs to be kept cool, which limits its use in low- and middle-income countries, and, until recently, it was thought that only trained personnel could give intramuscular injections. Consequently, administration of misoprostol, a synthetic prostaglandin that has effects similar to those of oxytocin, has been proposed as an alternative way to prevent postpartum hemorrhage in resource-limited settings. Misoprostol is stable at room temperature, and because it can be given sublingually (beneath the tongue), it acts very quickly. However, the comparative efficacy of sublingual misoprostol and intramuscular oxytocin for the prevention of postpartum hemorrhage has not been established. Here, the researchers undertake a double-blinded, double-dummy randomized controlled non-inferiority trial to compare sublingual misoprostol and intramuscular oxytocin for the prevention of postpartum hemorrhage in Uganda, a country where there are more than 5,500 maternal deaths every year. A randomized controlled trial compares the outcomes of individuals assigned to different interventions through the play of chance. In a double-blinded trial, neither the researchers nor the participants know who is receiving which intervention. In this particular trial, double-blinding is achieved by giving a dummy (placebo) sublingual pill to the women assigned to the oxytocin group and a dummy injection to the women assigned to the misoprostol group, as well as their assigned treatments. A non-inferiority trial investigates whether one treatment is not worse than another treatment.

**What Did the Researchers Do and Find?:**

The researchers measured blood loss over the first 24 hours after delivery in 1,140 women admitted to a regional referral hospital in Uganda. The women were given either sublingual misoprostol or intramuscular oxytocin at the currently recommended doses, along with matching placebos, immediately after the birth of their child. Postpartum hemorrhage (defined as the loss of more than 500 ml of blood within 24 hours of delivery; the trial's primary outcome) occurred in 28.6% and 17.4% of the women in the misoprostol and oxytocin groups, respectively (an absolute risk difference of 11.2%). Severe postpartum hemorrhage (loss of more than 1,000 ml of blood within 24 hours of delivery) occurred in 3.6% and 2.7% of participants in the misoprostol and oxytocin groups, respectively, but this difference was not statistically significant (it could have happened by chance). On average, women given misoprostol had lost slightly more blood by two and 24 hours after delivery than those given oxytocin. There were no significant differences between the groups in terms of death, the need for blood transfusion, or the use of additional drugs to prevent blood loss, but women given misoprostol experienced shivering and fever more often than those given oxytocin.

**What Do These Findings Mean?:**

In their study protocol, the researchers specified that sublingual misoprostol would be deemed non-inferior to intramuscular oxytocin if the absolute risk difference for postpartum hemorrhage between the misoprostol and oxytocin treatment groups was less than 6% (the “non-inferiority” margin). These findings therefore indicate that sublingual misoprostol given at the recommended dose is inferior to intramuscular oxytocin for the prevention of postpartum hemorrhage in women undergoing an uncomplicated birth at a regional referral hospital in Uganda. Although several aspects of this study may affect the accuracy and generalizability of its findings (for example, women at high risk of birth complications were excluded from the study), the researchers conclude that oxytocin should remain the preferred agent for the prevention of postpartum hemorrhage where it is available. However, they note, sublingual misoprostol remains important for the prevention of postpartum hemorrhage where oxytocin is unavailable or its administration is not feasible.

**Additional Information:**

Please access these websites via the online version of this summary at http://dx.doi.org/10.1371/journal.pmed.1001752.

- The United Nations Children's Fund (UNICEF) provides information on maternal mortality; “Trends in Maternal Mortality: 1990 to 2013” is a recent WHO/UNICEF/UNFPA/World Bank publication that provides up-to-date information on maternal mortality worldwide
- The World Health Organization provides information on maternal health (in several languages)
- The Postpartum Hemorrhage Prevention and Treatment Website provides a forum for information sharing and learning between organizations and individuals working on the prevention and treatment of postpartum hemorrhage in developing countries; the website includes basic information about postpartum hemorrhage and links to additional resources
- “Veil of Tears” contains personal stories (including stories about postpartum hemorrhage) from Afghanistan about loss in childbirth
- “Maternal Death: The Avoidable Crisis” is a briefing paper published by Médecins Sans Frontières in 2012
- More information about this trial is available

## Introduction

Of the estimated 287,000 maternal deaths worldwide, 85% occur in low- and middle-income countries [1]. In Uganda, the maternal mortality ratio is among the highest in the world, estimated at over 360 for every 100,000 women [2] and claiming the lives of over 5,500 mothers annually. Twenty-five percent of these deaths occur because of postpartum hemorrhage (PPH) within 24 h of delivery [3].

Oxytocin, a hormone that stimulates uterine contractions and limits uterine bleeding after birth, is the standard of care for prevention of PPH during the third stage of labor [4]. The use of oxytocin in low-income countries, however, has historically been limited by a number of factors including a perceived requirement for administration by skilled personnel, cold chain storage, and a requirement for sterile syringes and needles [5],[6]. Recent work has begun to challenge these limitations, as exemplified by effective administration of oxytocin by lay community health officers during home births [7].

Misoprostol, a synthetic prostaglandin with uterotonic properties, has been proposed as an alternative strategy for prevention of PPH in settings where oxytocin use is not feasible. It has important advantages over oxytocin, including the potential for oral administration and a long shelf life at room temperature [8]. Moreover, misoprostol can be administered sublingually, enabling a more rapid onset of action and greater bioavailability by avoiding first-pass metabolism [9]. These characteristics have led civil society organizations in Uganda to champion increased accessibility and use of misoprostol as a complementary drug to oxytocin in prevention of PPH [6]. Yet despite these advantages, sublingual misoprostol remains a second-line option to injectable uterotonics according to most recommending agencies [4],[10] because of insufficient [11] or conflicting [12] evidence about its efficacy in the active management of the third stage of labor. Although prior studies have compared injectable oxytocin with misoprostol [11], the comparative efficacy of sublingual misoprostol versus oxytocin remains largely unknown because prior studies have focused on oral administration of misoprostol by less skilled birth attendants [13],[14], evaluated oral as opposed to sublingual administration of misoprostol [15], or evaluated suboptimal doses of either oxytocin [16], other injectable uterotonics, or misoprostol [17]–[19].

We performed a double-blind, double-dummy randomized controlled non-inferiority trial comparing sublingual misoprostol versus oxytocin at a publically funded regional referral hospital in rural, southwestern Uganda. We aimed to elucidate the comparative benefit of oxytocin versus sublingual misoprostol, at the World Health Organization recommended dose of 600 µg [4], for prevention of PPH during active management of uncomplicated labor at a large referral hospital in a resource-limited setting. We hypothesized that sublingual misoprostol would be non-inferior to oxytocin for prevention of primary PPH.

## Methods

### Ethics Statement

This study was approved by the Institutional Review Committee of Mbarara University of Science and Technology and the Uganda National Council for Science and Technology. Trial registration at ClinicalTrials.gov (NCT01866241) was completed approximately halfway through the study. The delayed registration was due to the prolonged leave of the single ClinicalTrials.gov administrator at the principal investigator's institution.

### Study Design and Setting

We conducted a double-blind, double-dummy randomized controlled non-inferiority trial at Mbarara Regional Referral Hospital, a publically funded teaching hospital in southwestern Uganda serving ten districts with a population of over 5 million people. The hospital employs seven obstetricians and 22 midwives. Hospital staff perform over 10,000 deliveries annually. Prior to the study, we performed a retrospective review of hospital records to estimate rates of attended births, PPH, and maternal mortality. During that review, we counted 9,027 births over a 10-mo period. During that period, 2,979 mothers (33%) were recorded to have had PPH, and 11 mothers died during admission because of complications of PPH (0.12% of mothers in the review period).

### Participants and Recruitment

Midwife research assistants (MRAs) screened laboring mothers in early active labor on arrival to the prenatal ward. Eligibility criteria were (1) age above 18 y, (2) 38–41 wk of amenorrhea, and (3) anticipated uncomplicated vaginal delivery as assessed by hospital staff. The exclusion criteria were (1) confirmed intrauterine fetal death, (2) self-reported maternal heart disease, (3) current diagnosis of severe malaria or acute bacterial infection, (4) multiple pregnancy, (5) induced or augmented labor, (6) elective cesarean section, (7) antepartum hemorrhage, (8) reported hypersensitivity to prostaglandins, and (9) altered cognitive status as assessed by MRA. MRAs obtained informed consent from all eligible participants after the birth was predicted to be an uncomplicated vaginal delivery. An MRA trained in human participant research conducted informed consent procedures with eligible mothers in the local language in a private area of the hospital. Only mothers in the early stages of labor (less than 6 cm dilation) were approached. All consenting participants gave written informed consent, or for those who could not write, a thumbprint was made on the consent form.

### Randomization, Blinding, and Medicine Preparation

A study biostatistician generated a randomization list with a block size of ten, totaling 570 participants in each group. The list was shared only with the study clinical pharmacist, who prepared the study drugs and placebos. Each participant received a treatment (600 µg of misoprostol or 10 IU of oxytocin) and placebo (injection of 1 ml of sterile water or three pills containing maize starch, methyl hydroxybenzoate, and magnesium stearate) within 1 min of birth. An independent clinical pharmacist at Mbarara University of Science and Technology prepared the corresponding treatments and placebos. Misoprostol 200-µg pills (Cytotec Searle, United Kingdom) were procured from Laborex Uganda. Oxytocin (10 IU/1 ml) was procured from Joint Medical Store (Kampala, Uganda). Before the use of the medications, we performed bioequivalence testing for both active interventions at the Ugandan National Chemotherapeutics Research Laboratory. Bioequivalence for misoprostol ranged from 95.8% to 99.8%, and for oxytocin ranged from 94.7% to 103.5%. To achieve blinding of the participants and assessors, both inactive agents were manufactured and packaged to resemble actual study medicines in terms of shape, size, and color by Kampala Pharmaceuticals Industries (Uganda).

### Study Procedures

MRAs received opaque envelopes with affixed study codes, containing both an injection (1 ml of oxytocin 10 IU or its placebo) and three pills (misoprostol 600 µg or its placebo), which were given intramuscularly and sublingually, respectively, within 1 min of delivery. Delayed cord clamping was preferred, and the placenta was delivered by controlled cord traction or manually if not delivered within 30 min postpartum, as per Ugandan national clinical guidelines [20]. Further care was provided by the hospital clinical care team in collaboration with MRAs in accordance with national guidelines, which recommend administration of a repeat dose of parenteral oxytocics along with bladder emptying, management of lacerations, and uterine massage if bleeding persists. All mothers were monitored for a minimum of 24 h postpartum.

### Study Measures

A blood sample for complete blood count was drawn immediately after admission and again prior to hospital discharge, or before blood transfusion. MRAs recorded vital signs, duration of second and third stages of labor, secondary use of open-label uterotonics, placental retention, requirement for blood transfusion, and side effects, using a standardized data collection form. After the baby was born, the amniotic fluid was drained immediately. A clean plastic sheet specifically designed and piloted to collect blood for this trial was placed under the mother's buttocks during and after the third stage of labor. Blood was drained into a calibrated container to improve accuracy in blood loss measurement [21],[22]. All mothers were given preweighed standard sanitary pads to place in the perineum at all times. These pads were changed and weighed hourly for the first 6 h, and then every 6 h until 24 h postpartum. Blood loss was estimated as 1 ml per gram of weight of the pad after subtracting the dry pad weight, as previously described [22]. This estimated blood loss was added to the volume of blood from the plastic sheet. To improve consistency in estimation of blood loss, standardized electronic scales were used to weigh soiled sanitary pads.

### Study Outcomes

Our primary outcome was primary PPH, defined as maternal loss of blood ≥500 ml within 24 h of birth, as conventionally defined [4]. Secondary outcomes included the following: (1) death; (2) severe PPH, defined as maternal blood loss ≥1,000 ml within 24 h of birth; (3) changes in red cell indices during hospitalization, defined as (a) postpartum hemoglobin <100 g/l, (b) >10% decrease between pre- and postpartum hemoglobin, (c) mean postpartum hemoglobin, and (d) mean postpartum hematocrit; (4) mean measured blood loss at 1, 2, and 24 h postpartum; (5) placental retention; (6) requirement for blood transfusion (which is indicated per clinical protocol at the study site for mothers with a hemoglobin <100 g/l and/or severe pallor); (7) requirement for additional management of PPH, including therapeutic uterotonic drugs or surgical or radiological procedures; and (8) duration of the third stage of labor. The outcomes of blood loss at 1 and 2 h postpartum were post hoc analyses added to enable direct comparisons with other studies, which have often used those end points. All patients were assessed for continued blood loss at 2 h postpartum, when a second blood sample was drawn from participants for complete blood count, blood type, and cross-matching. To avoid measurement bias, we used the values from this measurement to calculate changes in hemoglobin and hematocrit levels for women who received a subsequent blood transfusion. We also compared the safety profile of both treatment groups, including observed rates of shivering, nausea and vomiting, fever >37.5°C within 24 h of delivery, self-reported headache, diarrhea, abdominal afterpains, and the use of analgesics in the postpartum period.

### Sample Size and Statistical Analysis

We followed CONSORT guidelines for conducting and reporting a non-inferiority study [23]. We designed the non-inferiority study with a 6% absolute risk difference as our non-inferiority margin (Δ_NI_). We selected the non-inferiority margin of 6% based on prior data comparing oxytocin with placebo for active management of labor that demonstrated a 50% relative reduction in the rate of PPH with oxytocin versus placebo [24]. Assuming a predicted incidence of PPH of 14% in mothers treated with prophylactic oxytocin, as reported previously by a well-powered clinical trial [15], a non-inferiority margin of 6% would correspond to an upper bound of PPH incidence of 20% among mothers treated with misoprostol. We chose this upper bound of non-inferiority so that a 20% PPH rate in the misoprostol arm would be similar to the rate for women treated with oxytocin and likely superior to predicted rates of PPH in women not receiving treatment [25]. Allowing for a two-sided type I error of 5%, we planned enrollment of 1,140 mothers to enable 90% power to demonstrate non-inferiority between groups. We compared dichotomous outcomes between study groups by estimating crude relative risks (RRs) with 95% confidence intervals, and testing for differences between treatment groups. We estimated *p*-values with chi-squared tests using a level of significance of 0.05. We also tested for the significance of absolute risk differences using the *Z*-test of proportions. For continuous outcomes, we estimated *p*-values using Student's *t* tests. All primary and secondary outcomes were analyzed using intention-to-treat analyses (although no participants were misallocated treatment) [23]. As per the revised CONSORT guidelines for reporting randomized trials [26], we assessed for sub-group effects for the following characteristics by testing the significance of interaction terms in a multivariable logistic regression model: (1) age (18–35 y and >35 y), (2) parity (1, 2–4, and ≥5), (3) birth weight (<2,500 g, 2,500–3,449 g, and ≥3,500 g), (4) placental weight (<0.8 and ≥0.8 kg), (5) any perinatal surgical procedure (episiotomy and/or perineal tear and no episiotomy or perineal tear), (6) admission hemoglobin (<120 g/l and ≥120 g/l), and (7) body mass index (<25 and ≥25 kg/m^2^). Finally, although our study was fully randomized, we noted differences between treatment groups in the proportion of women with the following characteristics: presence of perineal tears, requirement for episiotomy, and parity. As such, we performed post hoc analyses to assess for confounding by fitting multivariable logistic regression models to assess for differences in our by-treatment estimates after adjustment for these characteristics. All statistical analyses were performed using STATA version 12.0 (StataCorp, College Station, Texas, US). An independent data safety monitoring board composed of members at the Mbarara University of Science and Technology, Makerere University, and Mbarara Regional Referral Hospital reviewed preliminary results at 50% (570) and 75% (855) of projected enrollment, as specified in the protocol, and recommended continuing study procedures.

## Results

**Figure 1:**
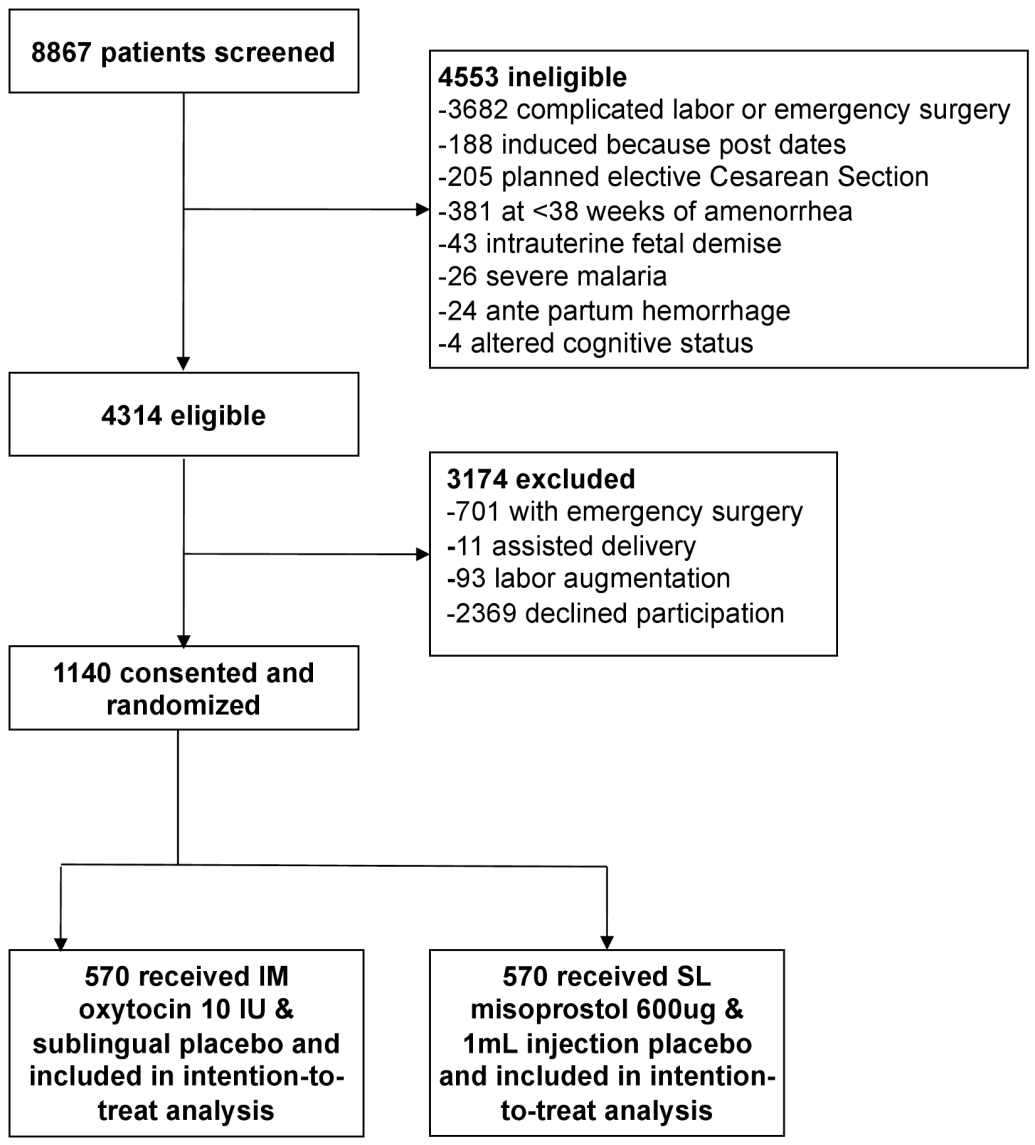
Trial profile. IM, intramuscular; SL, sublingual.

Of 8,867 mothers screened for eligibility from 23 September 2012 to 9 September 2013, 4,314 were eligible. A total of 2,369 (55%) declined participation in the study (Figure 1), and 1,140 were enrolled, received a randomized treatment, and completed study procedures. Demographic and clinical characteristics were similar between the two treatment groups (Table 1). Primary PPH occurred in 163 (28.6%) participants in the misoprostol group and 99 (17.4%) participants in the oxytocin group (RR 1.64, 95% CI 1.32 to 2.05, *p*<0.001; absolute risk difference 11.2%, 95% CI 6.44 to 16.1; Table 2), corresponding to a number needed to treat of nine (meaning that nine women would need to be treated with oxytocin instead of misoprostol to prevent one case of PPH). The absolute risk difference between the two groups failed to meet the pre-specified non-inferiority margin of 6%. In stratified analyses to assess for differences in our primary outcome within sub-groups, none of the sub-group-by-treatment interaction terms was significant (Table 3). With the exception of women with parity ≥5, all of the point estimates favored oxytocin. Thus, while the study was not powered to estimate effects within sub-groups, our results do not suggest differential effects of treatment within specific sub-groups of mothers.

**Table 1:** Baseline demographic and clinical characteristics by treatment group.

Characteristic	Misoprostol Group (*n* = 570)	Oxytocin Group (*n* = 570)
**Mean age (years) (SD)**	29.3 (3.4)	29.7 (3.1)
**Educational attainment less than secondary, ** ***n*** ** (percent)**	361 (63.3%)	352 (61.8%)
**Parity, ** ***n*** ** (percent)**		
1	249 (43.7%)	219 (38.4%)
2–4	273 (47.9%)	286 (50.2%)
≥5	47 (8.3%)	64 (11.3%)
**Mean gestational age (SD)**	39.2 (0.8)	39.3 (0.8)
**Mean weight of placenta (SD)**	0.6 (0.1)	0.6 (0.1)
**Mean duration between previous and current pregnancy** * **(years) (SD)**	3.7 (2.3)	3.9 (2.2)
**Mean birth weight (kg) (SD)**	3.1 (0.4)	3.2 (0.5)
**Perineal tear, ** ***n*** ** (percent)**	80 (14.0%)	59 (10.4%)
**Episiotomy, ** ***n*** ** (percent)**	177 (31.1%)	144 (25.3%)
**Mean pre-delivery Hb (g/l) (SD)**	131 (14)	132 (13)
**Pre-delivery Hb, ** ***n*** ** (percent)**		
<120 g/l	85 (14.9%)	76 (13.3%)
<100 g/l	21 (3.7%)	20 (3.5%)
<80 g/l	0	0
**History of PPH, ** ***n*** ** (percent)**	65 (11.4%)	66 (11.6%)
**Mean hematocrit at admission (** ***n*** ** = 943) (SD)**	39.3 (4.0)	39.6 (4.1)
**Prenatal visits (** ***n*** ** = 1,132), ** ***n*** ** (percent)**		
0	0 (0.0%)	2 (0.4%)
1–3 visits	83 (14.7%)	97 (17.2%)
>3 visits	483 (85.3%)	467 (82.5%)
**History of home birth** * **, ** ***n*** ** (percent)**	127 (39.6%)	142 (40.5%)
**Mean duration of second stage of labor (minutes) (SD)**	13.7 (6.2)	13.4 (6.1)

Total mothers *n* = 570 unless otherwise specified.

*Excludes primigravid mothers.

Hb, hemoglobin; SD, standard deviation.

**Table 2:** Primary and secondary outcomes by treatment group.

Category	Outcome	Misoprostol Group (*n* = 570)	Oxytocin Group (*n* = 570)	RR (95% CI)	*p*-Value	Absolute Risk Difference (95% CI)
**Primary outcome**	**Blood loss ≥500 ml at 24 h**	163 (28.6%)	99 (17.4%)	1.64 (1.32 to 2.05)	<0.001	11.2 (6.39 to 16.07)
**Secondary outcomes**	**Blood loss ≥1,000 ml**					
	24 h	20 (3.6%)	15 (2.7%)	1.33 (0.69 to 2.58)	0.391	0.9 (−1.12 to 2.88)
	2 h	18 (3.2%)	14 (2.5%)	1.29 (0.65 to 2.56)	0.473	0.7 (−2.62 to 1.22)
	1 h	11 (1.9%)	10 (1.8%)	1.10 (0.47 to 2.57)	0.826	0.1 (−1.41 to 1.72)
	**Blood loss ≥500 ml**					
	2 h	89 (15.6%)	57 (10.0%)	1.56 (1.14 to 2.13)	0.005	5.6 (1.75 to 9.48)
	1 h	53 (9.3%)	35 (6.1%)	1.51 (1.00 to 2.28)	0.046	3.2 (0.06 to 6.25)
	**>10% Hb drop** β	139 (24.4%)	114 (20.0%)	1.22 (0.98 to 1.52)	0.075	4.4 (−0.65 to 9.35)
	**Maternal Hb at discharge** β					
	<120 g/l	204 (35.8%)	166 (29.1%)	1.23 (1.04 to 1.44)	0.016	6.7 (1.24 to 12.09)
	<100 g/l	48 (8.4%)	49 (8.6%)	0.98 (0.67 to 1.43)	0.916	0.2 (−3.23 to 3.43)
	<80 g/l	4 (0.7%)	9 (1.6%)	0.44 (0.14 to 1.43)	0.163	0.9 (−0.35 to 2.22)
	**Receipt of blood transfusion**	7 (1.2%)	16 (2.9%)	0.44 (0.18 to 1.06)	0.058	1.7 (−0.05 to 3.21)
	**Use of additional uterotonics**	47 (8.2%)	31 (5.4%)	1.51 (0.98 to 2.35)	0.062	2.8 (−0.13 to 5.75)
	**Retained placenta**	5 (0.9%)	4 (0.7%)	1.25 (0.34 to 4.63)	0.738	0.2 (−1.07 to 1.04)
	**Mean blood loss (ml) (SD)**					
	24 h	484.7 (213.3)	432.8 (203.5)	N/A	<0.001	N/A
	2 h	341.5 (206.2)	304.2 (190.8)	N/A	0.002	N/A
	1 h	223.2 (183.1)	193.4 (159.7)	N/A	0.004	N/A
	**Mean postpartum hematocrit (** ***n*** ** = 943) (SD)** β	0.361 (0.046)	0.366 (0.048)	N/A	0.117	N/A
	**Mean postnatal Hb (g/l) (SD)** β	120 (14)	121 (15)	N/A	0.074	N/A
	**Mean duration of third stage of labor (min) (SD)**	4.4 (2.0)	4.4 (1.9)	N/A	0.823	N/A
	**Maternal death**	0	0	0	—	—
**Safety endpoints**	**Headache**	10 (1.8%)	11 (1.9%)	0.91 (0.39 to 2.13)	0.829	0.1 (−0.04 to 1.07)
	**Nausea/vomiting**	138 (24.2%)	86 (15.1%)	1.60 (1.26 to 2.05)	<0.001	9.1 (4.54 to 13.71)
	**Fever >37.5°C**	53 (9.3%)	12 (2.1%)	4.42 (2.39 to 8.18)	<0.001	7.2 (5.05 to 9.38)
	**Shivering (observed)**	321 (56.4%)	168 (26.5%)	1.91 (1.65 to 2.21)	<0.001	29.9 (24.41 to 35.47)
	**Diarrhea**	6 (1.1%)	2 (0.4%)	2.98 (0.60 to 4.72)	0.155	0.7 (−0.27 to 1.67)
	**Afterpains**	132 (23.2%)	163 (28.6%)	0.81 (0.66 to 0.99)	0.034	5.4 (0.41 to 10.39)

Data are *n* (percent) unless otherwise indicated.

β Pre-transfusion hemoglobin/hematocrit levels used.

Hb, hemoglobin; N/A, not applicable; SD, standard deviation.

**Table 3:** Maternal baseline sub-groups by treatment group with PPH.

Sub-Group (*n*)	Misoprostol Group (*n* = 570)	Oxytocin Group (*n* = 570)	RR (95% CI)	*p*-Value	*p*-Value for Interaction Term
**Age**					
18–35	156/549 (28.4%)	92/547 (16.8%)	1.69 (1.34 to 2.12)	<0.001	0.417
>35	7/21 (33.3%)	7/23 (30.4%)	1.10 (0.46 to 2.60)	0.837	
**Parity**					
1	79/249 (31.7%)	41/219 (18.7%)	1.69 (1.21 to 2.36)	0.001	0.203
2–4	75/273 (27.5%)	44/286 (15.4%)	1.79 (1.28 to 2.49)	0.001	
≥5	9/47 (18.8%)	14/64 (21.5%)	0.88 (0.41 to 1.85)	0.726	
**Birth weight**					
<2,500 g	4/23 (17.4%)	5/22 (22.7%)	0.77 (0.24 to 2.48)	0.655	0.269
2,500–3499 g	118/423 (27.9%)	62/411 (15.1%)	1.85 (1.40 to 2.44)	<0.001	
≥3,500 g	41/124 (33.1%)	32/137 (23.4%)	1.42 (0.96 to 2.10)	0.081	
**Placenta weight**					
≥0.8 kg	24/61 (39.3%)	12/55 (21.8%)	1.80 (1.00 to 3.25)	0.042	0.606
<0.8 kg	139/509 (27.3%)	87/515 (16.9%)	1.62 (1.27 to 2.05)	<0.001	
**Surgical procedures**					
Episiotomy/perineal tear	73/204 (35.8%)	32/167 (19.2%)	1.87 (1.30 to 2.68)	<0.001	0.231
No episiotomy or tear	90/366 (24.6%)	67/403 (16.6%)	1.48 (1.11 to 1.96)	0.006	
**Admission Hb**					
<120 g/l	26/85 (30.6%)	21/76 (27.6%)	1.11 (0.68 to 1.80)	0.680	0.120
≥120 g/l	137/485 (28.2%)	78/494 (15.8%)	1.79 (1.40 to 2.29)	0.000	
**Body mass index**					
≥25 kg/m^2^	92/311 (29.6%)	58/308 (18.8%)	1.57 (1.18 to 2.10)	<0.002	0.688
<25 kg/m^2^	71/259 (27.4%)	41/262 (15.6%)	1.56 (1.11 to 2.20)	0.009	

**Figure 2:**
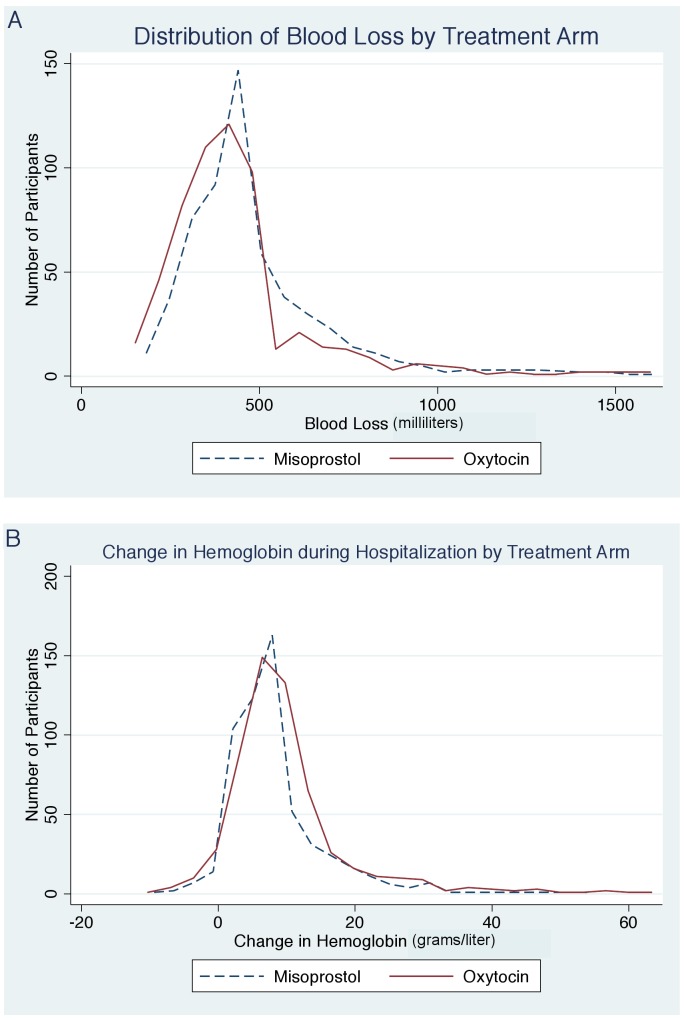
Distribution of blood loss and hemoglobin change by treatment arm. (A) Distribution of blood loss by treatment arm. (B) Change in hemoglobin during hospitalization by treatment arm.

We found a benefit for oxytocin versus misoprostol in terms of measured blood loss at 1 and 2 h postpartum (Table 2). The measured blood loss distribution was skewed to the right for both those receiving misoprostol (range 46.7–1,557.4 ml; median 457.6 ml) and those receiving oxytocin (range 28.1–1,617.8 ml; median 410.4 ml) (Figure 2). Importantly, there were no deaths in either group, and we found no statistically significant difference in the incidence of severe PPH at 24 h postpartum, which occurred in 20 (3.6%) participants in the misoprostol group and 15 (2.7%) in the oxytocin group (RR 1.33, 95% CI 0.69 to 2.58, *p* = 0.391; absolute risk difference 0.9%, 95% CI −1.12 to 2.88). There was also no difference in the rate of severe PPH between groups as estimated at 1 and 2 h postpartum. More mothers in the misoprostol group than in the oxytocin group received additional open-label oxytocin (*p* = 0.062). The number of mothers requiring and receiving a blood transfusion was higher in the oxytocin group than in the misoprostol group, but the difference did not reach statistical significance (2.9% versus 1.2%, *p* = 0.058). There were also no significant differences in hemoglobin change (*p* = 0.075), mean postpartum hemoglobin (*p* = 0.074), rate of retained placenta (*p* = 0.378), or duration of the third stage of labor (*p* = 0.823) (Table 2).

While we performed a randomized control trial and any differences in baseline characteristics occurred by chance, we did detect baseline differences in parity, requirement for episiotomy, and requirement for repair of perineal tears between study groups (Table 1). We assessed for bias from differences in baseline characteristics between groups by fitting multivariable logistic regression models. In these models, we found no meaningful difference in the odds ratio of PPH for misoprostol versus oxytocin after adjustment for parity, perineal tears, and requirement for episiotomy (adjusted odds ratio 0.54, 95% CI 0.41 to 0.72, *p*<0.001).

Side effects were more common in the misoprostol group than in the oxytocin group. A greater proportion of women in the misoprostol group than in the oxytocin group experienced moderate to severe shivering (56.4% versus 26.5%, RR 1.91, 95% CI 1.65 to 2.21, *p*<0.001), nausea and vomiting (24.2% versus 15.1%, RR 1.60, 95% CI 1.26 to 2.05, *p*<0.001), and temperature >37.5°C (9.3% versus 2.1%, RR 4.42, 95% CI 2.39 to 8.18, *p*<0.001). Afterpains were more common in the oxytocin group than in the misoprostol group (*p* = 0.036). No differences were found between the misoprostol and oxytocin groups in the rates of diarrhea (*p* = 0.155) or headache (*p* = 0.829).

## Discussion

We demonstrated that sublingual misoprostol is inferior to oxytocin for prevention of primary PPH in women undergoing uncomplicated vaginal deliveries at a publically funded regional referral hospital in southwestern Uganda. We found a 64% increased risk of primary PPH (measured blood loss ≥500 ml at 24 h) and an absolute risk increase of 11.2% with misoprostol versus oxytocin. We also found a 33% higher rate of severe PPH (measured blood loss ≥1,000 ml) in the misoprostol group, although this difference was not statistically significant. There were no maternal deaths in either group. The rates of secondary outcomes, including mean postpartum hemoglobin, requirement for additional uterotonics, hemoglobin changes, blood transfusion, duration of third stage of labor, and retained placenta were similar in both groups. While not statistically significant, we did observe a lower absolute rate of blood transfusion and proportion of women with postpartum hemoglobin <80 g/l in the misoprostol group.

Our data contribute to a complex array of data on optimal prevention of PPH in the third stage of labor in resource-limited settings. Like many prior studies [11], we found a modest benefit for oxytocin over misoprostol. In summary, we estimate that only nine women (95% CI 6 to 16) would need to be treated with oxytocin instead of misoprostol to prevent one case of primary PPH. On the other hand, our study was restricted to relatively healthy women without significant co-morbidities, and we detected no deaths at the time of discharge in either group. Moreover, we detected a difference in mean measured blood loss of only 29 ml, 37 ml, and 52 ml at 1, 2, and 24 h postpartum, respectively, and no differences in other secondary outcomes, including severe PPH, death, hemoglobin changes, or receipt of a blood transfusion. Our data therefore signal that, among relatively healthy women undergoing uncomplicated labor, oxytocin provides modest benefit over sublingual misoprostol for prevention of PPH generally, and should be the preferred agent where feasible and available. However, although we did not include a placebo arm in our trial, the lack of significant difference in other clinically significant outcomes between the treatment groups also offers promising preliminary data that sublingual misoprostol at a dose of 600 µg is likely to be of important benefit where oxytocin is unavailable. If and whether select populations gain preferential benefit from oxytocin over misoprostol remains an important question for further investigation.

Our results are largely consistent with prior studies comparing misoprostol with oxytocin for prevention of PPH. Only one prior trial (*n* = 100) specifically compared sublingual misoprostol at the routinely used dose of 600 µg to oxytocin at a dose of 10 IU, and found a lower mean blood loss with oxytocin [27]. A similar study comparing lower dose sublingual misoprostol with oxytocin 10 IU found a non-significant decrease in blood loss with oxytocin at 1 h postpartum [28]. In contrast, another study also comparing low-dose sublingual misoprostol 400 µg with oxytocin 10 IU found misoprostol more effective in prevention of PPH at 2 h postpartum [21]. Importantly, that study used a powdered sublingual formulation of misoprostol and was unintentionally unblinded because of lack of proper placebos [29]. Finally, a small study (*n* = 60) compared sublingual misoprostol 600 µg to syntometrine in place of oxytocin, and found no difference in PPH between the two groups [30]. Unlike these prior studies, our study was powered to demonstrate non-inferiority of a clinical outcome (or lack thereof) for sublingual misoprostol at a recommended dose of 600 µg versus conventional intramuscular oxytocin 10 IU in prevention of primary PPH, as defined by WHO (measured blood loss ≥500 ml at 24 h postpartum).

While we acknowledge that a recent large systematic review comparing misoprostol with injectable uterotonics in the management of the third stage of labor has been conducted [11], the prior evidence related to use of sublingual misoprostol 600 µg has been largely limited by variability of comparator uterotonics, dosing of study drugs, and heterogeneity of outcome definitions. For example, in 72 trials discussed in this review (*n* = 52,678), only 663 women received sublingual misoprostol, and only 60 women did so at the recommended dose of 600 µg [30]. In summary, our data are the first, to our knowledge, powered to evaluate whether the routinely used and recommended dosing of sublingual misoprostol (600 µg) is non-inferior to oxytocin 10 IU for the outcome of PPH.

Importantly, and in contrast to our study, prior studies have demonstrated a significantly higher risk of both PPH (≥500 ml) and severe PPH (≥1,000 ml) when oral misoprostol is used versus oxytocin [11]. For example, a large WHO-lead trial [16] documented a one percentage point difference in blood loss of ≥1,000 ml at 1 h postpartum for misoprostol versus oxytocin (4% versus 3%). This is in contrast to the minimal difference (0.1%) we detected in measured blood loss at 1 h postpartum (1.9% versus 1.8%). Although we did not directly compare use of oral with sublingual misoprostol, our results—specifically the relatively small differences detected in mean measured blood loss, hemoglobin change, and rates of PPH and severe PPH—support preferential consideration of sublingual misoprostol over the oral route of administration. A potential alternative to sublingual administration of misoprostol in prevention of PPH may be a powdered formulation of sublingual misoprostol, which has shown superior efficacy compared to oxytocin [21]. A theoretical advantage of sublingual misoprostol could be improved bioavailability gained by evading first-pass metabolism [9].

Another potential explanation for differences between our study and prior data, which have shown larger effect sizes for differences between prostaglandins and oxytocin, is our exclusion of women with cesarean deliveries and multiple pregnancies. Our selection criteria could underestimate true differences in bleeding risk in the general population, and specifically in higher risk women.

We found increased rates of side effects with misoprostol versus oxytocin, as has been previously reported [11]. Although misoprostol-related shivering is typically considered a nonserious side effect, prior studies have reported fever [31],[32], secondary psychological effects including anxiety, and perceptions of lack of body control [33]. Other concerns have included resultant delays in blood transfusion, and mimicry of postpartum infection resulting in unnecessary antibiotic administration, although this may be uncommon [15].

The benefit of oxytocin over sublingual misoprostol for prevention of PPH in this trial was seen across most sub-groups. Effect sizes appeared smaller in certain sub-groups, for example, women with admission hemoglobin less than 120 g/l, those older than 35 y, those giving birth to infants with a birth weight less than 2,500 g, and those with parity greater than four, which corroborates prior work demonstrating increased risk of PPH with advanced maternal age, with anemia [34],[35], in multiparous women, and in women with infants of low birth weight [36]. Although there were observed differences in point estimates of PPH incidence in these sub-groups, we found no significant differences in the effect of the treatment across these categories.

Our study had a number of strengths. All study investigators and clinical staff were blinded to treatment allocation. We used placebos for both oxytocin injection and misoprostol pills. Although blinding might have been unmasked, particularly by known side effects (e.g., shivering), we found similar benefit for oxytocin in a sub-analysis of women without documented shivering (RR 1.62, 95% CI 1.18 to 2.23, *p* = 0.003). We performed the study in a prototypical, publically funded and operated hospital in a rural setting with an active maternity unit, subject to the standard limitations of public sector health care facilities in the region. As such, the study has great potential for generalizability to similar settings.

Our study had some important limitations. We observed a decrease in maternal PPH from 33% to 17% and in maternal mortality from 0.12% to 0% from the pre-study period to the study period, suggesting either presence of strict exclusion criteria, inaccurate estimation of blood loss in the pre-study period, or possibility of a Hawthorne effect, which might have resulted from use of trained MRAs in the study. We also noted clustering of blood loss measurement between 400 and 500 ml in both groups. We suspect this was an observer bias stemming from the prespecified dichotomous outcome of ≥500 ml over 24 h. While this might have diminished the overall outcome incidence, our blinding procedures make it unlikely that measurement error would bias our estimates.

Another limitation of our study was the observed rate of eligible participants declining participation (54.9%). A review of stated reasons for declining participation revealed that most (97%) participants who declined were disinterested in participating in a research study, which was perhaps not unexpected given that most women were presenting in active labor. A recent survey study on the ward (Dr. Lenard Abesiga, personal communication, 1 November 2012) demonstrated that most mothers on the maternity ward in this hospital (92%) have little or no knowledge of medicines administered during labor. Nonetheless, the high declination rate might introduce a selection bias towards relatively healthy women.

### Conclusion and Recommendations

We found that sublingual misoprostol 600 µg is inferior to oxytocin 10 IU for prevention of primary PPH during active management of the third stage of labor among women undergoing uncomplicated delivery in a rural referral hospital in southwestern Uganda. Severe PPH was rare in our study population, and we detected no significant difference between those receiving sublingual misoprostol versus oxytocin (RR 1.33, 95% CI 0.69 to 2.58, *p* = 0.391). There were also similar rates of changes in postpartum hemoglobin, duration of the third stage of labor, requirement for additional uterotonics, and requirement for a blood transfusion. These data demonstrate that, in settings where it is available, oxytocin should remain a preferred agent for prevention of PPH. However, sublingual misoprostol appears to maintain an important role for prevention of severe PPH and other complications of PPH where oxytocin is not available, and reinforces the array of available interventions for reducing maternal morbidity and mortality.

Further work should help clarify whether and in which sub-populations preferential use of oxytocin might have the highest impact. This is particularly important in resource-limited settings where storage and availability of oxytocin remains a major challenge. Additionally, further evaluation of the actual and perceived barriers to oxytocin use for prevention of PPH in resource-limited settings will help improve its availability and use in such settings.

## Supporting Information

Checklist S1 CONSORT checklist.
(DOC)

Data S1 Dataset.
(CSV)

Text S1 Trial protocol.
(PDF)

## Abbreviations

MRA: midwife research assistant
PPH: postpartum hemorrhage
RR: relative risk
SD: standard deviation

## References

[1] World Health Organization, United Nations Children's Fund, United Nations Population Fund, World Bank (2012) Trends in maternal mortality: 1990–2010. WHO, UNICEF, UNFPA and The World Bank estimates. Geneva: World Health Organization.

[2] United Nations Department of Economic and Social Affairs Population Division (2013) World population prospects: the 2012 revision. New York: United Nations Department of Economic and Social Affairs Population Division.

[3] Uganda Ministry of Health (2010) Clinical guidelines for prevention and treatment of post partum haemorrhage using misoprostol. Kampala: Uganda Ministry of Health.

[4] World Health Organization (2012) WHO recommendations for the prevention and treatment of postpartum haemorrhage. Geneva: World Health Organization. 23586122

[5] Prata N, Bell S, Weidert K (2013) Prevention of postpartum hemorrhage in low-resource settings: current perspectives. Int J Womens Health 5: 737–752 10.2147/IJWH.S51661 24259988 PMC3833941

[6] Atukunda EC, Brhlikova P, Agaba AG, Pollock AM (2013) Registration, procurement, distribution, and use of misoprostol in Uganda: an interview-based observational study. Lancet 382: 10.

[7] Stanton CK, Newton S, Mullany LC, Cofie P, Tawiah Agyemang C, et al (2013) Effect on postpartum hemorrhage of prophylactic oxytocin (10 IU) by injection by community health officers in Ghana: a community-based, cluster-randomized trial. PLoS Med 10: e1001524. 24130463 10.1371/journal.pmed.1001524 PMC3794862

[8] Tang OS, Gemzell-Danielsson K, Ho PC (2007) misoprostol: pharmacokinetic profiles, effects on the uterus and side-effects. Int J Gynaecol Obstet 99 (Suppl 2) S160–S167. 17963768 10.1016/j.ijgo.2007.09.004

[9] Katzung GB (2010) Basic principles of pharmacology. In: Katzung GB, Masters SB, Trevor AJ, editors. Basic and clinical pharmacology, 12th edition. New York: McGraw-Hill Medical.

[10] International Federation of Gynecology and Obstetrics (2012) Prevention of postpartum hemorrhage with misoprostol. Int J Gynaecol Obstet 119: 213–214 10.1016/j.ijgo.2012.09.002 23022109

[11] Tuncalp O, Hofmeyr GJ, Gulmezoglu AM (2012) Prostaglandins for preventing postpartum haemorrhage. Cochrane Database Syst Rev 8: CD000494 10.1002/14651858.CD000494.pub4 PMC7043277 22895917

[12] Chu CS, Brhlikova P, Pollock AM (2012) Rethinking WHO guidance: review of evidence for misoprostol use in the prevention of postpartum haemorrhage. J R Soc Med 105: 336–347 10.1258/jrsm.2012.120044 22907551 PMC3423133

[13] Derman RJ, Kodkany BS, Goudar SS, Geller SE, Naik VA, et al (2006) Oral misoprostol in preventing postpartum haemorrhage in resource-poor communities: a randomised controlled trial. Lancet 368: 1248–1253 10.1016/S0140-6736(06)69522-6 17027730

[14] Mobeen N, Durocher J, Zuberi N, Jahan N, Blum J, et al (2011) Administration of misoprostol by trained traditional birth attendants to prevent postpartum haemorrhage in homebirths in Pakistan: a randomised placebo-controlled trial. BJOG 118: 353–361. 21176086 10.1111/j.1471-0528.2010.02807.x PMC3041931

[15] Gulmezoglu AM, Villar J, Ngoc NT, Piaggio G, Carroli G, et al (2001) WHO multicentre randomised trial of misoprostol in the management of the third stage of labour. Lancet 358: 689–695. 11551574 10.1016/s0140-6736(01)05835-4

[16] Baskett TF, Persad VL, Clough HJ, Young DC (2007) Misoprostol versus oxytocin for the reduction of postpartum blood loss. Int J Gynaecol Obstet 97: 2–5 10.1016/j.ijgo.2006.12.016 17321529

[17] Vaid A, Dadhwal V, Mittal S, Deka D, Misra R, et al (2009) A randomized controlled trial of prophylactic sublingual misoprostol versus intramuscular methyl-ergometrine versus intramuscular 15-methyl PGF2alpha in active management of third stage of labor. Arch Gynecol Obstet 280: 893–897. 19277690 10.1007/s00404-009-1019-y

[18] Vimala N, Mittal S, Kumar S (2006) Sublingual misoprostol versus oxytocin infusion to reduce blood loss at cesarean section. Int J Gynaecol Obstet 92: 106–110. 16343498 10.1016/j.ijgo.2005.10.008

[19] Vimala N, Mittal S, Kumar S, Dadhwal V, Mehta S (2004) Sublingual misoprostol versus methlergometrine for active management of third stage of labor. Int J Gynaecol Obstet 87: 1–5. 15464767 10.1016/j.ijgo.2004.05.016

[20] Uganda Ministry of Health (2010) Uganda clinical guidelines: national guidelines on management of common conditions. Kampala: Uganda Ministry of Health.

[21] Bellad MB, Tara D, Ganachari MS, Mallapur MD, Goudar SS, et al (2012) Prevention of postpartum haemorrhage with sublingual misoprostol or oxytocin: a double-blind randomised controlled trial. BJOG 119: 975–982 10.1111/j.1471-0528.2012.03341.x 22703421

[22] Hoj L, Cardoso P, Nielsen BB, Hvidman L, Nielsen J, et al (2005) Effect of sublingual misoprostol on severe postpartum haemorrhage in a primary health centre in Guinea-Bissau: randomised double blind clinical trial. BMJ 331: 723 10.1136/bmj.331.7519.723 16195287 PMC1239973

[23] Piaggio G, Elbourne D, Pockock SJ, Evans SJ, Altman DG, et al (2012) Reporting of noninferiority and equivalence randomized trials: extension of the CONSORT 2010 statement. JAMA 308: 2594–2604. 23268518 10.1001/jama.2012.87802

[24] Westhoff G, Cotter AM, Tolosa JE (2013) Prophylactic oxytocin for the third stage of labour to prevent postpartum haemorrhage. Cochrane Database Syst Rev 10: CD001808 10.1002/14651858.CD001808.pub2 24173606

[25] Nordstrom L, Fogelstam K, Fridman G, Larsson A, Rydhstroem H (1997) Routine oxytocin in the third stage of labour: a placebo controlled randomised trial. Br J Obstet Gynaecol 104: 781–786. 9236641 10.1111/j.1471-0528.1997.tb12020.x

[26] Altman DG, Schulz KF, Moher D, Egger M, Davidoff F, et al (2001) The revised CONSORT statement for reporting randomized trials: explanation and elaboration. Ann Intern Med 134: 663–694. 11304107 10.7326/0003-4819-134-8-200104170-00012

[27] Tewatia R, Rani S, Srivastav U, Makhija B (2014) Sublingual misoprostol versus intravenous oxytocin in prevention of post-partum hemorrhage. Arch Gynecol Obstet 289: 739–742. 24045979 10.1007/s00404-013-3026-2

[28] Chaudhuri P, Biswas J, Mandal A (2012) Sublingual misoprostol versus intramuscular oxytocin for prevention of postpartum hemorrhage in low-risk women. Int J Gynaecol Obstet 116: 138–142 10.1016/j.ijgo.2011.09.016 22100204

[29] Weeks A (2012) Commentary on “prevention of postpartum haemorrhage with sublingual misoprostol or oxytocin: a double-blind, randomised controlled trial”. BJOG 119: 982–983. 10.1111/j.1471-0528.2012.03341.x 22703421

[30] Lam H, Tang OS, Lee CP, Ho PC (2004) A pilot-randomized comparison of sublingual misoprostol with syntometrine on the blood loss in third stage of labor. Acta Obstet Gynecol Scand 83: 650. 10.1111/j.0001-6349.2004.00572.x 15225189

[31] Elati A, Weeks A (2012) Risk of fever after misoprostol for the prevention of postpartum hemorrhage: a meta-analysis. Obstet Gynecol 120: 1140–1148. 23090533 10.1097/aog.0b013e3182707341

[32] Durocher J, Bynum J, Leon W, Barrera G, Winikoff B (2010) High fever following postpartum administration of sublingual misoprostol. BJOG 117: 845–852 10.1111/j.1471-0528.2010.02564.x 20406228 PMC2878599

[33] Amant F (2001) The misoprostal third stage study: a randomised controlled comparison between orally administered misoprostol and standard management. A double-blind placebo controlled randomised trial of misoprostol and oxytocin in the management of the third stage labour. BJOG 108: 338. 10.1111/j.1471-0528.2001.00082.x 11281484

[34] Babinszki A, Kerenyi T, Torok O, Grazi V, Lapinski RH, et al (1999) Perinatal outcome in grand and great-grand multiparity: effects of parity on obstetric risk factors. Am J Obstet Gynecol 181: 669–674. 10486482 10.1016/s0002-9378(99)70511-9

[35] Tsu VD (1993) Postpartum haemorrhage in Zimbabwe: a risk factor analysis. Br J Obstet Gynaecol 100: 327–333. 8494833 10.1111/j.1471-0528.1993.tb12974.x

[36] Stones RW, Paterson CM, Saunders NJ (1993) Risk factors for major obstetric haemorrhage. Eur J Obstet Gynecol Reprod Biol 48: 15–18. 8449256 10.1016/0028-2243(93)90047-g

